# Financial Toxicity Survey in Patients With Gynecologic Cancer

**DOI:** 10.1097/og9.0000000000000081

**Published:** 2025-05-08

**Authors:** Alana Lanser, Indira Pachipala, Rachael Piver, Bunja Rungruang, Sharad Ghamande, Sharad Purohit, Katherine P. Richardson, Jessa Suhner, Robert Higgins, Marian Johnson, David P. Mysona

**Affiliations:** Georgia Cancer Center and the Department of Obstetrics and Gynecology, Medical College of Georgia at Augusta University, and the Center for Biotechnology and Genomic Medicine, Augusta University, Augusta, Georgia.

## Abstract

**OBJECTIVE:**

To describe the sensitivity, specificity, and positive and negative predictive values of the COST (Comprehensive Score for Financial Toxicity) survey for predicting whether patients would report altering spending habits, borrowing money, not filling prescriptions, or experiencing an inability to afford groceries secondary to cancer care costs in a population of patients with gynecologic cancer.

**METHODS:**

This was a single-institution, cross-sectional study of patients with gynecologic cancer within 5 years of initial diagnosis or disease recurrence. Patients completed a one-time COST survey and were screened for altering spending habits, borrowing money, not filling prescriptions, or experiencing food insecurity.

**RESULTS:**

A total of 323 patients consented and completed the survey; their mean age was 63 years, and 36.5% self-identified as Black. The mean COST score was 22. The COST score had an area under the curve value of 0.92 for predicting whether a patient would report altering spending habits, borrowing money, not filling prescriptions, or experiencing food insecurity. The cutoff COST score to maximize sensitivity and specificity was 26. At this cutoff, the score was 83.1% sensitive and 88.2% specific and had a positive predictive value of 92.9% and a negative predictive value of 73.7% for identifying those who would screen positive for reporting altering spending habits, borrowing money, delaying buying medications, or experiencing food insecurity.

**CONCLUSION:**

Our data indicate that the COST score, a measure of financial toxicity, is associated with social determinants of health in patients with gynecologic cancer. The COST score may help to predict additional needs outside of financial assistance.

## Abstract 2

This article describes financial toxicity among patients with gynecologic cancer and uses the COST (Comprehensive Score for Financial Toxicity) score to determine cost-coping strategies and side effects.

*Financial toxicity* is the negative economic effect and associated distress that patients experience secondary to the cost of treatment.^1^ Estimates indicate that at least 40% of patients with any cancer experience financial toxicity, and financial toxicity is more common in women and individuals from racial and ethnic minority groups.^2^ In addition, financial toxicity is associated with an increased risk of death and poorer quality of life for patients with cancer despite controlling for malignancy specific factors, including stage of disease.^3^ Intuitively, clinicians know that the high costs of treatment negatively affect patients, but although physicians readily discuss the side effects of chemotherapy and other treatment options with patients, there is still ambiguity regarding how to discuss the financial side of care. The term “financial toxicity” was not referred to in the literature until 2009, when it was deemed a side effect of cancer care, and then further defined and explored in 2013 by Zafar and Abernethy, who are credited with coining the term in relation to the effects that the cost of cancer care has on patients.^4^ As a result, risk factors for financial toxicity and how patients respond have only recently been examined with limited data available for patients with gynecologic cancer.^1,5,6^

The prevalence of financial toxicity in patients with gynecologic cancer specifically has been estimated to be as high as 50%, which is similar to or higher than for other malignancies, indicating that these patients may be at an even higher risk.^6–8^ In addition, younger age, lower income, and insurance differences are linked to experiencing financial toxicity in gynecologic cancer populations.^6–9^ When financial toxicity has been screened for, most prior work has used the COST (Comprehensive Score for Financial Toxicity)–Functional Assessment of Chronic Illness Therapy survey.^6,7,9^ This survey scores financial toxicity on a scale from 0 to 44 and categorizes patients as having no/mild (score 26 or higher), moderate (score 14–26), or severe (score lower than 14) financial toxicity. The relationship between COST score and food insecurity has not explicitly been investigated to the best of our knowledge. Prior studies have demonstrated that patients with no/mild, moderate, and severe financial toxicity may change their spending habits, borrow money, and not fill medications to cope with cancer costs.^7,8^ In addition, it is unclear what the optimum value of the COST score is to screen for that a patient may report these negative side effects. This is especially important because there are limited time and ability to screen patients for all associated social determinants of health in a single visit.

The present study was a cross-sectional study designed to describe the relationship between COST scores and a patient reporting changing spending habits, borrowing money, not filling medications, or experiencing difficulty affording groceries secondary to the cost of their cancer care. As of now, there is no connection between patients and social work within our practice at the Georgia Cancer Center unless directly sought out by a patient. Furthermore, many patients are not aware of the resources available to them or may be embarrassed to seek assistance. Administering the COST survey to each patient after identification of an optimal cutoff value of the COST score could aid in identifying patients who may benefit from referral to discuss additional social work services outside of financial assistance such as connections to local food pantries.

## METHODS

This study was a single-institution, cross-sectional study approved by the Augusta University IRB of patients with gynecologic cancer at the Wellstar Georgia Cancer Center, which is the primary site of a Minority/Underserved National Cancer Institute Center Community Outreach Research Program with a catchment area of approximately 800,000 patients. All patients with a diagnosis of gynecologic cancer within 5 years of initial diagnosis or disease recurrence were eligible. *Gynecologic cancer* was defined as a diagnosis of ovarian, endometrial, cervical, vaginal, or vulvar cancer. Patients with borderline tumors were not eligible for enrollment. Informed consent was obtained from all patients. Patient demographic and cancer-related information was extracted from the patient's medical record. Race was included as part of the study because previous studies have shown a significant association between non-White race and financial toxicity.^7,9^ The electronic medical record at our institution did not allow patients to differentiate between race and ethnicity. We therefore categorized race and ethnicity as a single categorical variable. The number of comorbidities was scored with the Charlson Comorbidity Index.^10^ Information on income, number of dependents, and employment status was obtained as part of the distributed survey.

Between March 2023 and October 2024, patients who met eligibility criteria as defined previously and consented to be part of the study completed a one-time COST–Functional Assessment of Chronic Illness Therapy. This time period chosen was a convenience sample. The COST score was developed by the Functional Assessment of Chronic Illness Therapy and the University of Chicago to measure financial distress associated with cancer care by asking patients questions of a subjective nature regarding how they feel about their financial situation and its relationship to their illness. It was subsequently validated in a study looking at patients with solid tumors of any origin.^2,9^ Patients were divided into no/mild (score 26 or higher), moderate (score 14–26), or severe (score lower than 14) financial toxicity on the basis of the COST score as described previously.^5,10^ Patients were asked four additional questions about objective behavioral changes to screen changing spending habits secondary to the cost of cancer care, borrowing money to cover cancer care, purposefully not filling or delaying prescriptions secondary to the cost of cancer care, and experiencing food insecurity secondary to costs associated with cancer care. The four additional questions were phrased as follows: 1) “Due to your cancer diagnosis, have you changed your spending habits?” 2) “Due to your cancer diagnosis, have you borrowed money?” 3) “Have you delayed or avoided buying medication due to cost?” 4) “Has your cancer diagnosis made it difficult to afford buying groceries?” Patients were given the options to choose “yes,” “no,” or “prefer not to answer” as their response to each question. The question design was structured after survey questions previously published in gynecologic oncology.^5,11^

All statistical analyses were performed with the R programming language and environment.^11^ Categorical variables were assessed with the multinomial Cochran–Armitage test with the multiCA package 1.1 and the Jonckheere–Terpstra test with the DescTools package 0.99.59.^12,13^ These tests were chosen because they allowed the comparison of categorical and continuous variables with the ordinal grouping of the COST score. For all analyses, *P*<.05 was considered statistically significant. Receiver operator characteristic curves and associated area under the curve (AUC) values were generated with the pROC package 1.18.5 in R.^14^ The optimal cutoff for the COST score was determined with the OptimalCutpoints package 1.1-5 to determine the cutoff score that would provide the optimal specificity and sensitivity for a patient screening positive.^15^ The caret package was used to generate confusion matrixes and associated sensitivity, specificity, positive predictive value, and negative predictive value.^16^

## RESULTS

A total of 323 patients completed a COST survey. The mean age was 62.7 years, and the majority of patients were either White (60.4%) or Black (36.5%) race. More than half of the patients reported incomes of less than $30,000 (31.1%) or $30,000 or greater but less than $60,000 (21.1%), and 14.9% reported and income $60,000 or greater but less than $90,000. Only 11.8% of our respondents reported an income of $90,000 or higher, and 21.1% did not report an income. A total of 120 patients (37.1%) reported that cancer affected their employment; 204 patients (63.2%) had government insurance; 175 (60.4%) had two or more dependents; and 222 (69.8%) scored 2 or greater on the Charlson–Deyo Comorbidity Index. In terms of cancer-specific information, the majority of patients (77.4%) were diagnosed with endometrial or ovarian cancer, almost half (47.5%) had stage III or IV disease, and 70.4% had received chemotherapy with or without radiation as the primary treatment modality. Of the entire cohort, 115 patients (36.9%) had recurrent disease at the time of the survey (Table 1).

**Table 1.** Characteristics of All Patients Who Were Screened for Financial Toxicity (N=323)

Characteristic	Value
Age (y)	62.71±13.93
Race and ethnicity	
Asian	4 (1.2)
Black	118 (36.5)
Hispanic	5 (1.5)
Multiracial	6 (1.9)
White	190 (58.8)
Income ($)	
Less than 30,000	101 (31.1)
30,000–60,000	68 (21.1)
60,000–90,000	48 (14.9)
More than 90,000	38 (11.8)
No response	68 (21.1)
Employment change	
No, I continue to work full-time	32 (9.9)
No, I did not work before my diagnosis	158 (48.9)
Yes, I work part-time now	15 (4.6)
Yes, I had to stop working and have not gone back	64 (19.8)
Yes, I had to take short-term leave	41 (12.7)
No response	13 (4.0)
Insurance	
Government	204 (63.4)
Private	108 (33.5)
Self-pay	10 (3.1)
No. of financial dependents	
1	115 (39.7)
2	111 (38.3)
3 or more	64 (22.1)
Comorbidities (CCI score)	
0–1	96 (30.2)
2–3	138 (43.4)
4 or more	84 (26.4)
Cancer type	
Cervical	55 (17.1)
Endometrial	139 (43.2)
Ovarian	110 (34.2)
Vaginal	7 (2.2)
Vulvar	11 (3.4)
Cancer stage	
1	131 (43.2)
2	28 (9.2)
3	97 (32.0)
4	47 (15.5)
Recurrence	
No	197 (63.1)
Yes	115 (36.9)
Treatment	
Chemotherapy with or without radiation	223 (70.4)
Observation	18 (5.7)
Radiation alone	19 (6.0)
Surgery alone	57 (18.0)
COST score	21.66±12.45
COST category	
No/mild	128 (39.6)
Moderate	96 (29.7)
Severe	99 (30.7)
Alter spending habits	
No	116 (35.9)
Prefer not to answer	12 (3.7)
Yes	195 (60.4)
Borrow money	
No	220 (68.1)
Prefer not to answer	14 (4.3)
Yes	89 (27.6)
Delay medication	
No	256 (79.3)
Prefer not to answer	3 (0.9)
Yes	64 (19.8)
Food insecurity	
No	214 (67.3)
Prefer not to answer	10 (3.1)
Yes	94 (29.6)

CCI, Charlson Comorbidity Index; COST, Comprehensive Score for Financial Toxicity.

Data are mean±SD or n (%).

The mean±SD value of the COST score was 21.66±12.45 for the entire cohort. The distribution of scores is shown in Appendix 1, available online at http://links.lww.com/AOG/E109. In terms of the categorical breakdown of the COST score, 39.6%, 29.7%, and 30.7% were categorized as having no/mild (score 26 or higher), moderate (score 14–26), or severe (score lower than 14) financial toxicity, respectively. When patients were asked about altering spending habits, borrowing money, delaying medications secondary to cost, and experiencing food insecurity, 60.4%, 27.6%, 19.8%, and 29.6%, respectively, reported “yes” to these questions (Table 1).

The association of demographic and cancer-related information with the experience of financial toxicity was explored in patients categorized as having no/mild, moderate, and severe financial toxicity as outlined in Table 2. Financial toxicity was more common in younger patients. Specifically, patients with severe toxicity had a mean age of 56.9 years compared with 60.9 years in those with moderate financial toxicity and 68.5 years in those with no/mild financial toxicity (*P*<.001). Race was also associated with financial toxicity (*P*=.003). White patients made up 70.3%, 50.0%, and 52.5% of patients in the no/mild, moderate, and severe financial toxicity groups, respectively. Black patients made up 27.3%, 44.8%, and 40.4% of patients in the no/mild, moderate, and severe financial toxicity groups (*P*=.003). In addition, lower income was associated with financial toxicity (*P*<.001). A greater number of patients in the severe financial toxicity group reported an income of less than $30,000 (41.4%) compared with those in the moderate (38.5%) and no/mild (18.0%) financial toxicity groups (*P*<.001). Insurance type also had a significant association with the experience of financial toxicity (*P*<.001). In the severe and moderate groups, 45.9% and 40.6%, respectively, more often had private insurance compared with only 18.8% in the no/mild financial toxicity group (*P*<.001). Number of financial dependents was also associated with financial toxicity, with patients in the severe (30.0%) and moderate (28.6%) groups being more likely to report living in households of three or more compared with the no/mild group (11.2%) (*P*=.004). The Charlson Comorbidity Index was associated with financial toxicity (*P*<.001). Patients in the no/mild group had higher Charlson Comorbidity Index scores compared with those in the moderate and severe financial toxicity groups (*P*<.001). There was a significant association in how patients in the three groups responded when asked about employment (*P*<.001). Patients in the no/mild group were more likely to report that they did not work before diagnosis (73.4%). In the severe financial toxicity category, 35.4% of patients stopped working because of their diagnosis and did not return compared with 19.8% in the moderate group and only 7.8% in the no/mild group.

**Table 2.** Association of Comprehensive Score for Financial Toxicity Categorical Groupings With Patient Demographic, Cancer-Specific, and Treatment-Related Factors

Characteristic	COST Category			*P*
	No/Mild (n=128)	Moderate (n=96)	Severe (n=99)	
Age (y)	68.54±12.03	60.90±14.06	56.94±13.30	<.001
Race and ethnicity				
Asian	0 (0.0)	1 (1.0)	3 (3.0)	
Black	35 (27.3)	43 (44.8)	40 (40.4)	.003
Hispanic	3 (2.3)	1 (1.0)	1 (1.0)	
Multiracial	0 (0.0)	3 (3.1)	3 (3.0)	
White	90 (70.3)	48 (50.0)	52 (52.5)	
Income ($)				
Less than 30,000	23 (18.0)	37 (38.5)	41 (41.4)	<.001
30,000–60,000	24 (18.8)	20 (20.8)	24 (24.2)	
60,000–90,000	34 (26.6)	5 (5.2)	9 (9.1)	
More than 90,000	25 (19.5)	9 (9.4)	4 (4.0)	
No response	22 (17.2)	25 (26.0)	21 (21.2)	
Employment change				
No, I continue to work full-time	8 (6.2)	12 (12.5)	12 (12.1)	
No, I did not work before my diagnosis	94 (73.4)	41 (42.7)	23 (23.2)	
Yes, I work part-time now	1 (0.8)	7 (7.3)	7 (7.1)	<.001
Yes, I had to stop working and have not gone back	10 (7.8)	19 (19.8)	35 (35.4)	
Yes, I had to take short-term leave	10 (7.8)	10 (10.4)	21 (21.2)	
No response	5 (3.9)	7 (7.3)	1 (1.0)	
Insurance				
Government	103 (80.5)	54 (56.2)	47 (48.0)	<.001
Private	24 (18.8)	39 (40.6)	45 (45.9)	
Self-pay	1 (0.8)	3 (3.1)	6 (6.1)	
No. of financial dependents				
1	51 (44.0)	33 (39.3)	31 (34.4)	.004
2	52 (44.8)	27 (32.1)	32 (35.6)	
3 or more	13 (11.2)	24 (28.6)	27 (30.0)	
Comorbidities (CCI score)				
0–1	18 (14.3)	35 (37.2)	43 (43.9)	<.001
2–3	62 (49.2)	42 (44.7)	34 (34.7)	
4 or more	46 (36.5)	17 (18.1)	21 (21.4)	
Cancer type				
Cervical	17 (13.3)	17 (17.9)	21 (21.2)	.066
Endometrial	62 (48.4)	35 (36.8)	42 (42.4)	
Ovarian	40 (31.2)	37 (38.9)	33 (33.3)	
Vaginal	6 (4.7)	1 (1.1)	0 (0.0)	
Vulvar	3 (2.3)	5 (5.3)	3 (3.0)	
Cancer stage				
1	54 (44.3)	43 (48.3)	34 (37.0)	.784
2	13 (10.7)	5 (5.6)	10 (10.9)	
3	36 (29.5)	30 (33.7)	31 (33.7)	
4	19 (15.6)	11 (12.4)	17 (18.5)	
Recurrence				
No	77 (62.1)	57 (62.6)	63 (64.9)	.669
Yes	47 (37.9)	34 (37.4)	34 (35.1)	
Treatment				
Chemotherapy with or without radiation	83 (65.9)	66 (70.9)	74 (75.5)	.322
Observation	7 (5.6)	6 (6.5)	5 (5.1)	
Radiation alone	10 (20.6)	5 (5.4)	4 (4.1)	

CCI, Charlson Comorbidity Index; COST, Comprehensive Score for Financial Toxicity.

Data are mean±SD or n (%) unless otherwise specified.

No cancer- or treatment-related factors were significantly associated with the experience of financial toxicity in our patient cohort. There was no statistical difference in cancer type (*P*=.066), cancer stage (*P*=.784), or cancer recurrence (*P*=.669). As shown in Table 2, there was no difference in financial toxicity when considering the primary treatment that patients received after their diagnosis or when comparing between patients actively on treatment and those not on treatment at the time of the survey (*P*=.322).

Patients who experienced financial toxicity reported altering spending habits, borrowing money, not filling or delayed filling of prescriptions, and experiencing food insecurity (Table 3). Among those with severe financial toxicity, 69.4% reported food insecurity compared with 25.8% of those with moderate financial toxicity and 1.6% of those with no/mild financial toxicity (*P*<.001). Patients with severe financial toxicity also had the highest rates of delaying or not filling medications (43.4%) compared with those with moderate (18.8%) and no/mild (2.3%) financial toxicity (*P*<.001). Nearly two-thirds of patients (64.6%) in the severe financial toxicity group reported having to borrow money to cope with cancer-related costs compared with 24.0% in the moderate and 1.6% in the no/mild (*P*<.001) financial toxicity groups. Alteration in spending habits was most common in the severe financial toxicity group at 93.9% compared with 74.0% in the moderate and 24.2% in the no/mild financial toxicity groups (*P*<.001). The distribution of COST scores for patients who answered “yes” to each respective question is shown in Appendix 2, available online at http://links.lww.com/AOG/E109.

**Table 3.** Association of Comprehensive Score for Financial Toxicity Categorical Groupings With Patient Responses to Social Determinants of Health–Related Screening Questions

Screening Question	COST Category			*P*
	No/Mild (n=128)	Moderate (n=96)	Severe (n=99)	
Alter spending habits				
No	93 (72.7)	19 (19.8)	4 (4.0)	<.001
Prefer not to answer	4 (3.1)	6 (6.2)	2 (2.0)	
Yes	31 (24.2)	71 (74.0)	93 (93.9)	
Borrow money				
No	124 (96.9)	66 (68.8)	30 (30.3)	<.001
Prefer not to answer	2 (1.6)	7 (7.3)	5 (5.1)	
Yes	2 (1.6)	23 (24.0)	64 (64.6)	
Delay medication				
No	124 (96.9)	76 (79.2)	56 (56.6)	<.001
Prefer not to answer	1 (0.8)	2 (2.1)	0 (0.0)	
Yes	3 (2.3)	18 (18.8)	43 (43.4)	
Experience food insecurity				
No	123 (96.9)	63 (67.7)	28 (28.6)	<.001
Prefer not to answer	2 (1.6)	6 (6.5)	2 (2.0)	
Yes	2 (1.6)	24 (25.8)	68 (69.4)	

COST, Comprehensive Score for Financial Toxicity.

Data are n (%) unless otherwise specified.

**Fig. 1.**
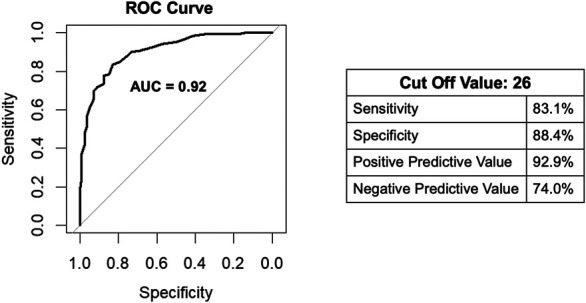
The COST (Comprehensive Score for Financial Toxicity) score receiver operator characteristic curve (ROC) for predicting whether a patient will alter spending habits, borrow money, delay medication, or have food insecurity. The area under the curve (AUC) value was 0.92. If the COST score was used to screen for the experience of one of these social determinants of health, a score of 26 would be 83.1% sensitive and 88.2% specific, with a positive predictive value of 92.9% and a negative predictive value of 73.7% for identifying those who would screen positive for altering spending habits, borrowing money, delaying buying medications, or experiencing food insecurity.

Given the prior data, the COST score was assessed as a continuous value for its ability to screen whether a patient would answer “yes” to altering spending habits, borrowing money, delaying or not obtaining medications, or experiencing food insecurity. The COST score had an AUC of 0.92 (95% CI, 0.89–0.95) for differentiating between those who would answer “yes” or “no” to these questions (Fig. 1). The receiver operator characteristic curve was used to determine the optimal cutoff that would optimize the sensitivity and specificity that a patient would screen positive for any one of the questions for altering spending habits, borrowing money, delaying or not filling medications, or experiencing food insecurity. The cutoff value was determined to be 26, which falls in the no/mild financial toxicity grouping. At a value of 26, the COST score was 83.1% sensitive and 88.42 specific with a positive predictive value of 92.9% and negative predictive value of 73.7 for identifying those who would screen positive for altering spending habits, borrowing money, delaying or not filling medications, or experiencing food insecurity because of their cancer care (Fig. 1).

*Lanser. Financial Toxicity in Gynecologic Oncology. O&G Open 2025*.

**Fig. 2.**
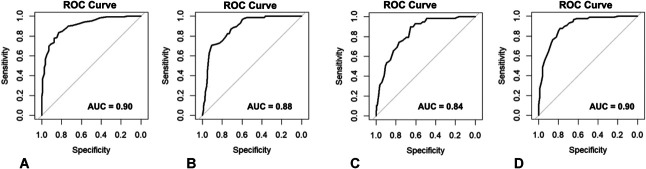
The receiver operator characteristic curves (ROCs) and associated area under the curve (AUC) values for the CIST (Comprehensive Score for Financial Toxicity) score for whether patients would report altering spending habits (AUC 0.90) **(A)**, borrowing money (AUC 0.88) **(B)**, delaying medication (AUC 0.84) **(C)**, or experiencing food insecurity (AUC 0.90) **(D)**.

When we examined each specific screening question, the COST score remained an excellent predictor of responses to individual questions. The AUC of the COST score was 0.90 (95% CI, 0.87–0.94) for predicting patients answering “yes” to altering spending habits (Fig. 2A), 0.88 (95% CI, 0.84–0.92) for patients reporting borrowing money (Fig. 2B), 0.84 (95% CI, 0.79–0.89) for patients reporting that they delay or do not fill medications because of cost (Fig. 2C), and 0.90 (95% CI, 0.86–0.94) for patients reporting food insecurity (Fig. 2D).

*Lanser. Financial Toxicity in Gynecologic Oncology. O&G Open 2025*.

The COST score cutoff of 26 was explored for its sensitivity, specificity, positive predictive value, and negative predictive value for predicting that a patient would respond “yes” to each respective question related to altering spending habits, borrowing money, delaying or not filling medication, and experiencing food insecurity (Table 4). At this cutoff, the COST score was 83.3% sensitive at identifying those who would alter spending habits, 97.7% sensitive at identifying those who would borrow money, 94.8% sensitive at identifying those who would report delaying or not filling medication, and 97.8% sensitive at identifying those who would report food insecurity secondary to the cost of their cancer treatments.

**Table 4.** Differences in Sensitivity, Specificity, Positive Predictive Value, and Negative Predictive Value for Individual Social Determinants of Health–Related Screening Questions at a Cutoff Value of 26

	Screening Question			
	Alter Spending Habits	Borrow Money	Delay Medication	Food Insecurity
Sensitivity	83.3	97.7	94.8	97.8
Specificity	82.9	58.3	51.1	59.4
PPV	88.8	49.1	32.5	51.5
NPV	75.4	98.4	97.5	98.4

PPV, positive predictive value; NPV, negative predictive value.

Data are %.

## DISCUSSION

Our study describes the COST tool, its association with financial toxicity in patients with gynecologic cancer, who is most at risk, and how patients cope with high costs of care. We also found that financial toxicity is prevalent in populations of patients with gynecologic cancer at a higher rate than demonstrated in previous studies and is predictive of patients choosing not to fill medications, taking on debt, and being unable to afford groceries.^7,8,17^ Furthermore, our results show that, although the COST score can vary from 0 to 44, a binary cutoff is capable of accurately identifying when patients may use cost-coping strategies, thus allowing efficient referral to necessary services. A patient's COST score was more than 94% sensitive for identifying patients who would report delaying medication, borrowing money, or experiencing food insecurity. Therefore, our work demonstrates that a binary cutoff of the COST survey can be used to efficiently rule out patients who do not need referral to financial navigators or other assistance programs while being confident we are not missing patients who need assistance. This will aid in streamlining referrals and simplifying the number of questionnaires needed to comprehensively assess their social needs. In addition, this study focuses on financial toxicity in patients receiving care at a Minority/Underserved National Cancer Institute Center Community Outreach Research Program.^9^

Our work is similar to prior publications demonstrating increasing rates of financial toxicity among younger patients, which has been proposed as being related to less savings, less access to social resources, and an inability to access certain government insurance plans.^1,3^ Our study demonstrated that a higher Charlson Comorbidity Index was associated with less financial toxicity. We suspect that this is secondary to patients 65 years of age and older qualifying for Medicare and concurrently having more comorbidities. Our data indicated a trend toward private insurance being associated with greater toxicity, which is in contrast to prior work.^7,8^ The high rate of private insurance in those with moderate and severe financial toxicity was unexpected and may be attributable to differences in deductibles between insurance plans. Similar to other studies, this study showed that Black patients experienced higher rates of financial toxicity. However, a major difference between our study and prior published works is the greater number of self-identifying Black patients.

More than 30% of patients in our study were of Black race compared with less than 5% of the study population in prior studies.^8,17,18^ Only one other study on financial toxicity in gynecologic oncology has had similar numbers of patients of Black race (27% compared with 36.5% in our study).^7^ One reason for the differences in racial composition across studies is likely the region of the country. Our study and the Esselen et al^7^ study included a cohort of patients recruited from the Southeastern United States, which has higher numbers of Black patients, compared with the Northeast, which is the region studied in most prior publications.^8,17–19^ These differences in study cohorts demonstrate the necessity of collaborations across institutions to improve our understanding of financial toxicity in diverse populations.

Our patient population also had the highest reported levels of financial toxicity to date with 60.4% of participants reporting moderate or severe financial toxicity compared with 20–47% reported in prior studies.^8,17^ It is noteworthy that 30.7% of patients in our study reported severe financial toxicity, which is double the 15% rate published in prior studies.^7^ The higher COST score in our study may be driven, in part, by only 11.8% of patients reporting an income higher than $90,000 in our study compared with more than 20–30% in prior studies.^8,17^ The increased rates of financial toxicity seen in our study highlight the importance of including centers specialized in serving diverse populations to appropriately estimate the rates of financial toxicity in patients with cancer.

Recognizing risk factors contributing to financial toxicity is important, but it is also crucial to comprehend how financial toxicity ultimately leads to worse health and quality-of-life outcomes for patients. Most investigators postulate that worse patient outcomes are related to the cost-coping measures used by patients.^3,17^ This behavior is why additional questions on common cost-coping measures were included in our study and is consistent with prior studies. Our study showed that many patients with high financial toxicity undertake cost-coping measures and need medication or food assistance. The COST score was remarkably able to identify those who would use cost-coping strategies in our population with an AUC of 0.92. This is reflected in the fact that the COST score at a cutoff of 26 was more than 94% sensitive for identifying patients who would report food insecurity or deferring medications. This finding supports the notion that screening for a single social determinant of health can be used to triage patients to referral to a multidisciplinary team capable of addressing all anticipated needs based on the single screening tool. This is essential because administering all screening tools at a single visit is not feasible. Our results are consistent with prior publications demonstrating that patients with high financial toxicity are at increased risk of using cost-cutting strategies to afford care.^7^ These data highlight the need for multidisciplinary teams to address financial toxicity that incorporate social work, financial navigators, pharmacy, and clinical staff. Future work will focus on whether this multidisciplinary approach mitigates financial toxicity and its downstream side effects. Furthermore, these data may help other institutions assess what resources need to be allotted when designing a program to address social determinants of health.

The present study has several limitations. This was a single-site study conducted at a Minority/Underserved National Cancer Institute Center Community Outreach Research Program. Including multiple institutions would make our results more generalizable. The study was cross-sectional, which prevents the capturing of financial toxicity changes over time with treatment. Because survey administration occurred at varying points in our patients' cancer journeys, there is likely some recall bias. In addition, we screened only for food insecurity in addition to the cost-coping strategies reported in prior publications but did not investigate other potential confounders such as number of prior cycles of chemotherapy. Future work could include administering the COST survey in combination with surveys such as the Accountable Health Communities Health-Related Social Needs Screening Tool, which screens for more than 20 social determinants of health. Adding this screening tool would provide a more comprehensive understanding of the interaction of financial toxicity and other social determinants of health such as housing insecurity. Strengths of our study include the large number of patients, a significant number of patients with recurrent or advanced stage disease, and the high participation of traditionally underrepresented populations. In addition, the use of the COST score and the known consistency of the performance of this tool add credence to the COST score becoming the standard tool for financial toxicity screening for the population with gynecologic cancer.^20^ Early monitoring of financial toxicity and additional social determinants of health could help to develop initiatives and resources to alleviate financial toxicity.
